# Ludwig von Bertalanffy's Organismic View on the Theory of Evolution

**DOI:** 10.1002/jez.b.22611

**Published:** 2015-02-27

**Authors:** Manfred Drack

**Affiliations:** 1 Department of Theoretical Biology, University of ViennaVienna, Austria; 2 Bertalanffy Center for the Study of Systems Science BCSSSVienna, Austria

## Abstract

Ludwig von Bertalanffy was a key figure in the advancement of theoretical biology. His early considerations already led him to recognize the necessity of considering the organism as a system, as an organization of parts and processes. He termed the resulting research program organismic biology, which he extended to all basic questions of biology and almost all areas of biology, hence also to the theory of evolution. This article begins by outlining the rather unknown (because often written in German) research of Bertalanffy in the field of theoretical biology. The basics of the organismic approach are then described. This is followed by Bertalanffy's considerations on the theory of evolution, in which he used methods from theoretical biology and then introduced his own, organismic, view on evolution, leading to the demand for finding laws of evolution. Finally, his view on the concept of homology is presented. *J. Exp. Zool. (Mol. Dev. Evol.) 324B: 77–90, 2015*. © 2015 The Authors. *Journal of Experimental Zoology Part B: Molecular and Developmental Evolution* published by Wiley Periodicals, Inc.

Ludwig von Bertalanffy (1901–1972) (Fig. 1) is widely known as the father of general system theory (GST). Some scholars are aware of his contributions to the concepts of open systems and steady state (flux equilibrium), and in some areas of research his growth equations are still being referred to today. Little, however, is known about Bertalanffy as one of the founding fathers of theoretical biology (Brauckmann, 2000; Pouvreau and Drack, 2007). He is also considered a forerunner of the organismic systems approach, which links his ideas to current evo-devo (Callebaut et al., 2007). Indeed, several issues that already concerned Bertalanffy are still being discussed today; and for many of them he provided new avenues of thinking. These issues include: evolutionary novelty, macroevolution beyond the explanatory framework of the modern synthesis, adaptationism, covariation, integration, and evolvability.

**Figure 1:**
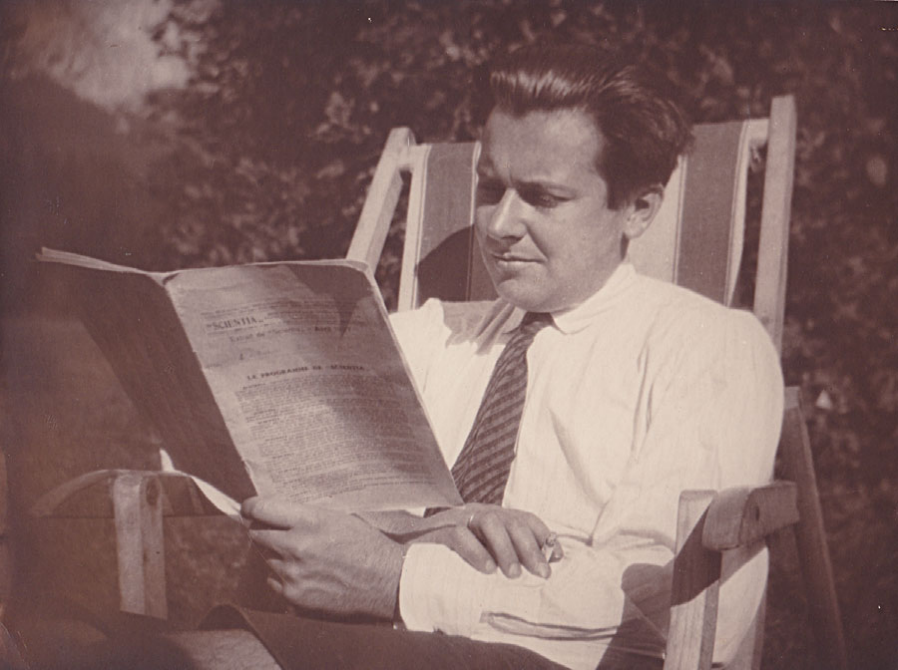
The young Ludwig von Bertalanffy in 1926. (© Bertalanffy Center for the Study of System Science, Vienna, Austria, BCSSS-Archiv: Ludwig von Bertalanffy Teilnachlass 2 [LvB-TN-2], Fotoalbum “Scrap Book,” Foto LvB, August 1926).

Gould and Lewontin (1979) criticized the predominant “adaptationist programme” in evolutionary thinking in the United States and England, which was based mainly on natural selection and treated the organism as an aggregate or sum of characters that can be changed almost completely independently of each other. In contrast to that, they re-introduced organismic notions from continental Europe. Although they did not consider Bertalanffy specifically, the latter contributed considerably to the foundations of such a scientific treatment of integrated systems that went beyond more or less plausible adaptationist explanations and did not explain all characters as adaptation. He was even able to account for constraints due to system conditions.

Bertalanffy's thinking is therefore also relevant for current (evolutionary) systems biology (e.g., Soyer, 2012) and related approaches (e.g., Winther, 2008). Very often only the GST book (Bertalanffy, 1969a) is referred to, leaving aside the substantial contributions that he made in biology while still in Europe. He started his career in Vienna and wrote his early works in German, which largely explains why they are not widely known.

This paper focuses on those rather unknown biological issues that Bertalanffy tackled. First, Bertalanffy's conception of theoretical biology is outlined. He was a key figure in establishing this discipline, which is meant to be useful for any biological question. It therefore needs to be discussed in some detail, also because it provides the logical and methodological basis for his critical approach to the prevalent theories of evolution. Theoretical biology is tightly related to the second part on the organismic approach or organismic biology. In this approach the system character of almost all biological entities is outlined. The approach that focuses on system features is again gaining momentum in current research (cf. complexity, network theory, etc.). Theoretical biology and the organismic approach form the basis of Bertalanffy's arguments regarding the theory of evolution—the third part. His critical investigations on theories of evolution—from Darwinism (here understood as Darwinism in the early 20th century, i.e., Darwin's account minus the inheritance of acquired characters) to the modern synthesis—are based on the methods that he developed in theoretical biology and the organismic approach. Lastly, the key concept of homology—also as seen from the theoretical and organismic perspective—is described.

Those who are interested in the historical issues of Bertalanffy's life, e.g., his ambivalent connection to the Vienna Circle (Hofer, 1996) are referred to the biography compiled by Pouvreau (2009). More about the many scholars who influenced Bertalanffy, from philosophy, biology, and other fields, can be found in Pouvreau (2013).

## THEORETICAL BIOLOGY

Theory in biology is not a modern invention, but can be traced back to ancient authors. Efforts to establish a field of research termed theoretical biology, however, only began some one hundred years ago. Authors such as Reinke (1901), Schaxel ('19), Uexküll ('20), Ehrenberg (1923) used the term *Theoretische Biologie* (*theoretical biology*) explicitly, but with different aims. Often, theoretical biology was associated with philosophy of nature (*Naturphilosophie*), epistemology and metaphysics rather than with the formation of scientific hypotheses and theories. Reinke's work appears as a vitalistic philosophy of nature. Uexküll's theoretical biology is in large parts pure philosophy. Ehrenberg attempts to deduce the phenomena of life from the principle of the necessity of death. All these approaches have their merits, but only a few of them—Schaxel probably being a case in point (cf. Reiss, 2007)—are directly interesting for biology as a natural science (Bertalanffy, 1932:3). Also Adolf Meyer-Abich's goal was to promote theoretical biology. For him, physical theory was a subset of biological theory (Meyer, 1934; Amidon, 2008). This view, however, was not shared by Bertalanffy.

Following Ehrenberg, Bertalanffy distinguishes two related, and not strictly divided areas (significations, *Sinn*) of theoretical biology. (1) The first area deals with the epistemological and methodological basis of biology. This includes, on the one hand, the rational or logical analysis of the basis of knowledge in biology (e.g., the problem of teleology, or the relationship between experiment and theory) to avoid contradictions. On the other hand, it includes the critique of concepts and methods. For instance, the term mechanism (*Mechanismus*), with its several different meanings, is analyzed in detail with the result that it is too ambiguous a term to be used when exact concepts are required (Bertalanffy, 1932:38ff,47; '37a:163f). The aim of such a procedure is a hypothesis-free and logically sound order of biological knowledge. (2) The second area is similar to that of physics, where the division between theoretical and experimental physics is well established (Bertalanffy, 1932:6). This area is about the formulation of basic natural laws that can be tested experimentally (Bertalanffy, 1930b:9). The descriptive biological facts have to be captured by overarching theories (Bertalanffy, 1928c:51f).

The reason why investigations in the first area are necessary—while theoretical physics does not deal with such issues—is that theoretical biology is not yet a fully developed science. Hence, theoretical biology has to deal with tasks that physics no longer has to (Bertalanffy, 1930b:11f). In physics, concepts such as force or energy are uses, and there is an agreement on how to use them. The question whether the explanations in biology can ultimately be reduced to concepts of physics and chemistry, however, cannot be decided yet, and appears to be rather unimportant (Bertalanffy, 1930b:26). Clearly, certain phenomena in biology cannot be described solely with concepts from physics or chemistry because they are not tackled by the latter disciplines. Among such concepts are: organization, teleology, hierarchy, or type (*Typus*).

Within the second area, Bertalanffy distinguishes between (2a) bottom-up (*von unten*) and (2b) top-down (*von oben*) theoretical biology (Bertalanffy, 1928c:58). (Note that this is unrelated with bottom-up and top-down in scientific explanations, where bottom-up refers to the level from the molecules upwards and top-down from higher levels, such as the organism, downwards.) The bottom-up approach consists of critically evaluating hypothesis and theories that were brought forward to explain certain phenomena of living organisms—demonstrating the valuable and discarding the useless. In research fields where almost all logically possible hypotheses were brought forward, it is reasonable to first analyze them before adding something new (Bertalanffy, 1928c:100; '30b:11f). The result of such an endeavor is an order of facts and theories. A paradigmatic example for such a theory screening is Bertalanffy's investigation of theories of development (Bertalanffy, 1928c:102ff). In that book—which was translated into English—he analyzes theories, spanning from crystal analogies to morphogenetic fields, and compares them logically and with regard to empirical evidence.

The top-down approach is characterized by deriving the single phenomena of life from a principle or theory (Bertalanffy, 1930b:13). At the time, the prominent examples were mechanism and vitalism (Bertalanffy, 1928c:100). Both, however, are problematic—a fact that Bertalanffy points out by means of logical and epistemological analysis (e.g., in Bertalanffy, 1932:47ff). Being aware of such difficulties, he cautiously builds a deductive approach himself. In order to do so he provides a definition of the organism as follows: “A living organism is a system consisting of a large number of different parts, organized in hierarchic order, in which a large number of processes are ordered in such a way that, through their continuous interactions within wide borders, with a continuous change of substances and energies, the system stays, even when disturbed from outside, in its own state, or it builds up that state, or these processes lead to the generation of similar systems” (Bertalanffy, 1932:83). The procedure would then be to deduce principles from the definition. By inserting specific conditions of certain phenomena into the definition, predictions can be made; these can then be compared to observations or experiments. This enables the theory to be tested (Bertalanffy, 1932:119). This definition may not be exhaustive because it neglects, for instance, the historical character of life, but it serves as a starting point (Bertalanffy, 1932:83ff; '52a:129). The organism-definition states the necessary and sufficient conditions for a natural object to be called living (Bertalanffy, 1952a:130).

The greater aim of theoretical biology is to arrive at laws of nature (Bertalanffy, 1928c:62,90ff). In this respect, theoretical physics serves as a role model. The exact details of the interplay between theories and laws are not provided by Bertalanffy, but he constantly refers to Viktor Kraft and his book on the basic forms of the scientific methods (Kraft, 1926; Bertalanffy, 1930b:12; '60:171). Important in this context is the difference between the hypothetico-deductive approach, which Bertalanffy strives for, and an inductive approach. To use a prominent example: the principles of mechanics, for instance of falling bodies without friction, cannot be inductively derived from empirical data (Bertalanffy, 1927b:661). Such principles are no empirical sentences because they are based on conditions that cannot be found empirically (Kraft, 1926:104). A similar logical character has to be achieved with laws in theoretical biology (Bertalanffy, 1928b:8). Mathematics is, however, not the only basis for a strict theory. The same logical approach can be used when deducing from idealized conditions, or conditions that abstract from certain disturbances (Bertalanffy, 1930b:12). The starting point is an intuitively derived hypothesis or theory. Empirical data can help on that path, but the aim is to deduce such data from the theory. The abundant amount of data has to be ordered by principles and laws; only then can one refer to this endeavor as science (Bertalanffy, 1932:2f). Laws are deductively derived sentences that follow from theoretical preconditions, whereas rules are inductive generalizations of empirical facts which are not a result of logical necessity (Bertalanffy, 1932:23).

To start with the aim to reduce the phenomena of the living to solely physics and chemistry would be a bias without any good reason and an unwarranted limitation. The peculiar features of living objects would then be at risk of being ignored (Bertalanffy, 1928c:52f). Such fundamental biases determine what problems an investigator sees. They influence the framing of the questions, the experimental procedure, the methods, and the type of explanation and theory that are provided for the investigated phenomena. “In fact, the dependence on prevailing attitudes of mind is the stronger the less it is felt” (Bertalanffy, 1952a:21). Bertalanffy points to an often false, or an outdated understanding of physics. Today, physicists abdicate causal explanations and rather search for connections between variables, from an initial to a final state. Similarly, with such an approach in biology one can also find regularities and hence laws, without the need to enter a meaningless debate on reductionism (Bertalanffy, 1932:5,38ff,67). The function of a theory is to provide a common explanation for a number of otherwise unrelated facts. An explanation is defined as the logical subordination of the specific under a more general (Bertalanffy, 1932:23f).

The knowledge about chemical reactions is necessary and important, but it does not answer the biological question of why this reaction occurs at this place and at this time in the organism and in general in a system-preserving manner (Bertalanffy, 1932:25). This serves as an example for why considerations beyond an atomistic approach—i.e., a mental division of the organism into single, independent parts—are necessary. Apparently this biased atomistic view prevented the development of a theoretical biology (Bertalanffy, 1930b:25f). The above argumentation makes it clear that a central issue of theoretical biology is the order or organization (Bertalanffy rarely distinguishes between the two terms) of the parts and processes in an organism. This problem was not tackled properly and therefore we lack theories and laws about it (Bertalanffy, 1932:122). Wholeness (*Ganzheit*) is the prime feature of life and must therefore be investigated with scientific methods (Bertalanffy, 1941:250; '49a:119).

## THE ORGANISMIC APPROACH

According to the analysis of Julius Schaxel—an important figure in establishing theoretical biology—the roots of the organismic approach can be traced back to Aristotle (Schaxel, 1922:236). Bertalanffy used the term organismic biology to characterize his own fundamental conceptions, and strove to free them from metaphysical connotations in order to arrive at a science that takes the peculiar features of organisms into account. The time when Bertalanffy started to develop his organismic approach was characterized by an ongoing dispute between mechanists and vitalists. His organismic approach was meant as a way of research beyond those two approaches. Bertalanffy quotes Schaxel, who summarized the problems of a mere mechanistic approach as follows: “What legitimizes the mechanist to talk about adaptation and purposefulness, about individuality, about the whole and its parts, about the unit, organization, harmony, regulation, activity, autonomy, and finally about the organism itself?” (Schaxel, 1922:158, cited in Bertalanffy, 1927a:211)

Vitalists pointed to the problems of the mechanistic approach, and Bertalanffy acknowledges Hans Driesch for introducing the concept of wholeness (*Ganzheit*) into science (Bertalanffy, 1928c:145). Wholeness stands in opposition to an atomistic approach with its predominant consideration of the parts. The experiments of Driesch on sea urchins—where he divided early stage embryos to yield single complete embryos—were crucial for this argument (cf. e.g., Driesch, 1908:59ff). Furthermore, Driesch's critique (since 1893) of the mechanistic approach is seen by Bertalanffy as the most important because it is logically the most consistent (Bertalanffy, 1952a:5). Hence, the neo-vitalists, like Driesch, were asking the important questions, but they only provided unsatisfactory answers, leaving the sphere of science to find themselves in metaphysical speculations.

The aim in Bertalanffy's organismic approach was to take the problems that were raised in that dispute seriously, but approach them with scientific means. Accordingly, organismic biology is a positivistic term and does not consider metaphysical concerns about a soul or entelechy (Bertalanffy, 1927a:230).

Organism is a central concept (*Urbegriff*) that cannot and does not need to be dissolved or reduced (Bertalanffy, 1928c:74). It is, however, important to arrive at a clear expression of this and other terms to make them operable (Bertalanffy, 1928c:142). The phenomena of life are always connected to an individual organism. A summative approach is therefore insufficient. Regulation is a striking example for this argument because it not only depends on the single parts, but also on the condition of the organism as a whole (Bertalanffy, 1929c:381f). Other phenomena such as metabolism, excitability, reproduction, or morphogenesis also cannot be explained sufficiently solely with analytic sciences (Bertalanffy, 1929b:391).

The organismic approach is a research program, a basis for proposing new questions, without providing any premature explanations—such as found in mechanicism and vitalism (Bertalanffy, 1932:V; '51a:8). In so proceeding, Bertalanffy is not dogmatic and also points to the merits of mechanistic or reductionistic explanations in cases where they appear to be appropriate. The starting point is not an either holistic or atomistic attitude, but rather facing the problem to then decide which approach can be used. In this manner the holistic or organismic approach is a working hypothesis with the aim of raising concrete questions and searching for solutions (Bertalanffy, 1937c:9; '52a:181). A one-sidedness in either direction has to be avoided, an attitude that Bertalanffy underlines when using a statement of Johann Wolfgang von Goethe as leitmotif: In looking at nature one has to pay attention to the one and the whole (*Müsset im Naturbetrachten immer eins wie alles achten*) (Bertalanffy, 1932:III).

In presenting general system theory, Bertalanffy provides an example to illustrate his approach that also holds for biology. When considering a set of parts, where each is connected to each other, all changes in one part affect the others. This system can develop differently. It is for instance conceivable that over time the interactions between certain parts become weaker or non-existent. In such a case isolated areas can appear that have only minor or even linear causal dependence of one part on the others. This is termed mechanization or progressive segregation (Bertalanffy, 1969aa:66ff). When applying this thought model to a developing embryo, for example, a primary connected system can be imagined that, as it grows, can segregate into different more or less independent regions. Hence, it is unsurprising that some sort of “mechanized” behavior can be found. This, however, is only one extreme of what is possible in the range from a completely connected system to isolated parts. Even though the organismic approach allows for different perspectives, for Bertalanffy the system is primary and mechanization secondary (Bertalanffy, 1932:99).

The central problem that Bertalanffy sees in (theoretical) biology is the organization of the parts and processes in such a way that they sustain the living whole, the organism. Knowing the single parts and processes in the finest detail that physics and chemistry provide—which is necessary—is, however, insufficient to explain their organization (Bertalanffy, 1932:VII,52; '37c:8; '52a:11,182). The laws for chemical reactions, for instance, are in principle insufficient to explain organized forms (*Gebilde*) (Bertalanffy, 1932:324; '52a:62). Thus, it is impossible to conclude a (vectorial) 3D form solely from scalar quantities, such as concentrations. The organization of all the physico-chemical processes is what distinguishes the living from the dead (Bertalanffy, 1934:347; '52a:13). The challenge that also arises here is that the whole organism shows properties that are absent in the isolated parts. As long as individual phenomena are investigated in isolation, no fundamental difference between the living and the non-living, and thereby no primary feature of life, will be found (Bertalanffy, 1952a:12). More recently, in the same line, Lewontin (2000) acknowledged that we cannot escape what he calls the dialectic between part and whole in biology.

There is a difference between summative and constitutive properties that should be noted. An example for a summative property is mass. Knowing the mass of part A and part B, one can easily calculate the mass of the two together. An example for constitutive properties are the chemical properties of isomers that consist of the same atoms. In this case it is necessary to know the arrangement of the atoms to potentially derive the properties of the molecule (Bertalanffy, 1945:6). An organism has many constitutive properties. Recent accounts still revolve around such ideas on emergence (e.g., Wimsatt, 2007:277–287), but hardly refer to the earlier thinker.

The teleological aspect of the central problem is evident in terms such as organ, function, organism, or pathology. This raises the question of the importance of each part or process for the preservation of the organism (Bertalanffy, 1929b:388; '30a:64). Furthermore, terms such as adaptation, regulation, regeneration, norm, or perturbation signify the preservation of a system state (*Systemzustand*) which we call life (Bertalanffy, 1929b:388). Two notes are necessary here. First, teleology was annotated with several different meanings, from an anthropomorphic psychic capacity of anticipating a goal and behavior to achieve this goal, to a “reverse” causal connection from a future state to a present state. Both these meanings are not applicable in biology. Second, Bertalanffy distinguishes different stages in biology. Broadly, there is a descriptive and an explanatory stage (Bertalanffy, 1932:9ff). Description is more important in biology than in other sciences due to the vast amount of different phenomena that have to be surveyed and ordered. In this stage, teleology plays a role because it is a true gain of knowledge when finding out what a sense organ or a thorn is good for (Bertalanffy, 1927a:235f; '28c:77). In the explanatory stage, however, teleology must not appear. How the single parts and processes are ordered to guarantee the preservation of the organism has to be causally explained (Bertalanffy, 1929b:384). Here he closely follows the clarification of Emil Ungerer—a botanist and philosopher of biology, who also published in Schaxel's book series on theoretical biology—on the “real contemplation of fitness (*echte Zweckbetrachung*)” and “preservation of the whole (*Ganzheitserhaltung*).” The organism and the processes in it seem to behave “as if” it was after some aim (Ungerer, 1922; Bertalanffy, 1930a:64; '32:17f, cf. also Kant, 2010:§68, Vaihinger, 1965). Seemingly teleological behavior then is a result of system laws. Bertalanffy suspects that the causal inner dynamic in the system as a whole approaches an equilibrium, which appears as if it were purposeful (Bertalanffy, 1930b:22; '32:13).

Parts can behave differently in isolation compared to parts in connection (Bertalanffy, 1957:8). This entails an epistemological problem. To know the behavior of one part, one would need to know the behavior of all the interactions it depends upon. This, however, leads in a circle because the same argument holds for all the behaviors of the other parts as well. The single processes in a system, therefore, can only be determined approximately. One way to handle this problem is to look for laws of the overall behavior (*Integralgesetze*) of the system without considering the single parts and processes (Bertalanffy, 1932:110). The laws of thermodynamics serve as a role model. Bertalanffy's growth equations are an example that such an approach is also advantageous in biology. In those equations, assimilation and dissimilation are connected to growth in a physiologically meaningful manner (for details see Pouvreau and Drack, 2007).

The dynamic character of biological systems is an issue that Bertalanffy underlines in the interaction among parts and processes, but also in the open system property of life: the constant replacement of all the parts while, as a whole, the organism remains relatively constant (Bertalanffy, 1932:116). This principle was already anticipated by Heraclitus and by Johannes Müller (Bertalanffy, 1937b:107). The dynamic equilibrium in an open system is different from the equilibrium in a closed system where no work can be done. Bertalanffy worked intensively on this issue and coined the term *Fließgleichgewicht* (steady state) (e.g., Bertalanffy, 1950; '53). The open system feature is a necessary condition for all life (for more details refer to Pouvreau and Drack, 2007; for the homeostatic properties of species see Rieppel, 2009).

Another important feature is the hierarchical order in living systems, which is connected to the principle of open systems. What in one system is an irreversible process (formation, aging, and death) can be viewed in the next higher level as a phase within a repeated dynamic (Bertalanffy, 1932:204). Everything is considered as dynamic. Structures which seem to be stable on one time scale appear as slow processes and functions as fast processes (Bertalanffy, 1932:249). This view also entails what Bertalanffy calls a dynamic morphology: it goes beyond a static description and connects the change of form with physiological processes. The task is to derive organic forms from the ordered play of “forces” that can be captured by quantitative laws (Bertalanffy, 1950:27; '52a:136). The hierarchy of processes reflects one particular meaning of the term hierarchy. In fulfilling his proposal of theoretical biology, Bertalanffy—based on Joseph H. Woodger—analyzed seven different meanings of this problematic term (Bertalanffy, 1952a:37ff). Hierarchy is apparently also present in the genome, and Bertalanffy points to a “rank-order of the genes, from genes that control single, often minute, characteristics to those that influence a larger number of characters in more or less extensive pleiotropism […], and finally to ‘superordinate' or ‘collective' genes (E. Fischer, Pfaundler) that direct the activity of numerous other genes” (Bertalanffy, 1952a:47). Considering hierarchies is also important because the state of a part can depend on the condition of higher levels or the organism; the performance of the parts is connected to the organism as a whole (Bertalanffy, 1934:347; '51b:231). This requires investigating all levels of organization. Research on one level cannot replace research on the higher levels (Bertalanffy, 1932:113). This view is also shared by recent researchers and is considered again as being important (cf. e.g., Noble, 2006). Note also that the analysis of the concept of hierarchy—division hierarchy in particular—by Woodger was transmitted via Bertalanffy to Willi Hennig. For Hennig's phylogenetic systematics this played an important role (Hennig, 1979:21, Rieppel, 2003)

The intrinsic or inner dynamics of “forces” within a system, rather than fixed structures, have to be investigated as the basis for the harmony and coordination of the processes (Bertalanffy, 1933b:258f). The inner condition or system conditions, the causal interconnections, are more important for an organism's performance than non-disruptive interaction with the environment. An organism's autonomy is based on the system conditions (Bertalanffy, '30/'31:394). The autonomy or activity of an organism is very important for Bertalanffy, and distinguishes his point of view from the conception of the organism as a reactive, stimulus-response machine driven solely by outside factors. This point is relevant not only for behavior and psychology (cf. Bertalanffy, 1929b), but also for his view on evolution, as discussed below.

Bertalanffy summarizes the leading principles of the organismic conception as follows: “*The conception of the system as a whole* as opposed to the *analytical* and *summative* points of view; the *dynamic conception* as opposed to the *static* and *machine-theoretical* conceptions; the consideration of the organism as a *primary activity* as opposed to the conception of its *primary reactivity*” (Bertalanffy, 1952a:18f). The attempt to overcome the mechanistic and vitalistic approaches yields the research program of an organismic biology, whereas the attempt to explain life is termed the system theory of life (Bertalanffy, 1932:80).

## EVOLUTION VIEWED FROM THEORETICAL BIOLOGY AND THE ORGANISMIC STANDPOINT

As a central theme, for Bertalanffy the organismic approach is relevant for all areas of biology, hence also for the theory of evolution. Even though he himself did not conduct deep research in this field, he arrived at theoretical and conceptual considerations of evolution which are hardly known today. His writings on evolution from the 1920s to the end of his life reflect his approach to theoretical biology: initiate with a critical investigation, then contribute to further scientific developments. Accordingly, the following discussion first points to his critique and then shows how Bertalanffy introduced the organismic approach to the theory of evolution.

### A Critical View on the Theories of Evolution

The logical and epistemological examination—as proposed in the program of theoretical biology—of the issue of evolution requires analyzing: the logical basis of what can be inferred from what premises, and what not; the concepts and assumptions; the accordance with empirical findings. The analysis of those different points is not always separable, and the following treatment is therefore not a strict sequence of analysis but rather a loose collection of issues that Bertalanffy thought about.

Bertalanffy's early critique on the theory of evolution, Darwinism in the early 20th century as it were, is in line with the dominant critique at the time. At that point, Darwinism as a theory was no longer to be taken seriously. Only later, when mutations were investigated in more detail, did Darwin become important again. According to Bertalanffy ('37c: 168; '69b:65), the classical and still best critique on Darwin is laid out by Eduard von Hartmann, starting in 1875 and refined in several writings (Hartmann, 1904). Sinai Tschulok (1922), who—according to Bertalanffy ('52b:161)—very well analyzed the logical structure of the concept of evolution, is also an important basis for critique on Darwinism.

Main points of critique already in Bertalanffy's early writings are (Bertalanffy, 1929a:101f): (1) Selection can only act on a character that already exists. No explanation for the essential problem of origin of a character is provided (Bertalanffy, 1928a:161). (2) For an organism it is more relevant to be in an advantageous situation or site (*Situationsvorteil*) than bearing small, even beneficial, modifications. (3) Can the higher degree of organization of higher taxa be explained in terms of (adaptive) utility? (4) Are small modifications useful, anyway? (5) Utility is a problematic concept because it is not the case that everything in an organism is useful. It suffices that characters are not disadvantageous. (6) How can random changes in individual parts give rise to the origin of harmoniously working organs?—The problem of co-adaptation.

Connections to current issues in evo-devo research are evident. Point (1) relates to evolutionary novelty, point (3) to macroevolution beyond the explanatory framework of population genetics, point (5) to adaptationism and point (6) to covariation, integration and evolvability.

Not all of the points are pursued in Bertalanffy's later writings (e.g., the advantageous situation)—and the subsequent text does not follow its sequence—, but the basic problems were already outlined. In the following further logical issues are discussed and the accordance with empirical findings is investigated.

In the early 20th century there was also critique on the connection of genetics and evolution. Many distinguished researchers criticized Darwin's ideas and particularly the concept of natural selection; among them were Hugo de Vries, Wilhelm Johannsen, William Bateson, Jan Paulus Lotsy, Richard Goldschmidt, and Thomas Hunt Morgan (Senglaub, 2000:558f). The question of how the constancy of genes over many generations can be reconciled with evolutionary change was raised (Bertalanffy, 1928c:33). Research on mutations, however, showed that this is not a major problem. Another issue with a long discussion was the reconciliation of a proposed gradual change in evolution with discrete entities of inheritance; it was actually solved by Udny Yule early on. This was pointed out by Conrad Hal Waddington in a meeting in Alpbach (Austria) where Bertalanffy also participated (Waddington, 1969:358). Another early question—following Johannes Paulus Lotsy—was whether it is justified to talk about a molecular group to cause a character. This refers to a critique of Mendelism and the assumed atomistic, independent genes acting on characters (Bertalanffy, 1928b:46).

From a logical point of view, Bertalanffy emphasized that the doctrine of descent (*Deszendenzlehre*) has to be distinguished from assumptions and potential explanations of evolutionary change (Bertalanffy, 1928a:160f). This point is also reflected by the fact that, in the second half of the 19th century, “evolutionism” became widely accepted while Darwin's explanations on how evolution proceeds were largely rejected (Bowler, 1988:47). The doctrine of descent is theoretically inferred from findings in morphology, embryology, biochemistry, geology, and appears as a logical postulate. With those who do not accept this, no further discussion about evolution is possible (Bertalanffy, 1937c:163). The doctrine of descent, however, is independent of the question what the tree of life looks like and the factors explaining evolution (Bertalanffy, 1929a:98ff). About the logical status he states: “Evolution is not a fact if we designate by this term something which is directly observable. Rather the concept of evolution is an extrapolation of certain facts the justification of which lies in the great amount of documentary evidence supporting it.” (Bertalanffy, 1952b:161) However, there are three basic facts of observation: “First, so far as experience goes, organisms arise only by way of reproduction from parental organisms. Second, only consanguinity produces organisms similar to their parents. Third, notwithstanding this similarity of parents and offspring, occasionally and quite frequently, variations from the parental type appear.” (Bertalanffy, 1952b:161f, following Tschulok, 1922:§32)

The extrapolation from observed facts requires certain assumptions for explaining and predicting even undiscovered facts (Bertalanffy, 1952b:162). These assumptions are, however, disputable. For instance, the statement that in billions of years nothing else has happened than what was seen in a few years in some laboratories appears to be a bold extrapolation (Bertalanffy, 1952b:164). The issue of “uniformitarianism” (i.e., the notion that causes and effects in the past and present are similar and that rates or intensities of causation are uniform through history) is still on the agenda of current evo-devo research (Love and Lugar, 2013); this also connects to considerations on the evolution of evolvability (Wagner, 2014:143).

The concept of adaptation, together with its relation to utility and purpose, needs to be analyzed and clarified. Different possible meanings of adaptation can be distinguished: first, the environment influences the organism; second, a functional improvement of the organism by itself; third, a purposeful psychic process (Bertalanffy, 1928c:26). Probably other meanings can be annotated as well. Nonetheless, the difference between a reactive, outside-directed or an active concept is apparent. A logical issue is the place of adaptation in an explanation. Goethe pointed to this problem with the statement: The ox does not have horns to poke, but rather because it has horns, it pokes (Bertalanffy, 1934:361). This is connected to the phenomenon of orthogenesis, i.e., evolutionary progress in a certain direction due to inner factors as reflected by allometric growth (Bertalanffy, 1951b:131). Such allometric patterns are also used by Gould and Lewontin (1979) to contrast adaptationist approaches. For Bertalanffy, who endorsed the concept of orthogenesis, it is not adaptation that brings forward such directional change, but rather because orthogenetic evolution occurred, certain performances can result (Bertalanffy, 1951a:341). Hence, a logical critique is towards the conclusion of selectionism that the direction of evolution is determined only by external factors. “But this conclusion does not follow from the premises. If selection represents a *necessary* condition of evolution, it does not follow that it indicates a *sufficient* condition.” (Bertalanffy, 1952a:93)

A logical reproach on Darwin's ideas—pointed out by Hermann J. Jordan—is that he worked inductively on changes of form and deductively transferred this to something completely different, namely the origin of systems. Hence, he ignored the difference between a property (e.g., length or color of an organ) and a part (e.g., an organ) (Bertalanffy, 1932:59f; '34:345). What was learned from species that differ in properties must not be directly transferred to explain the origin of new organs (Jordan, 1929:352).

An important logical issue is whether the empirical findings can be explained by the hypotheses on the causes. Bertalanffy acknowledges that phenomena on the micro-evolutionary scale can be explained by mutation, selection, and isolation, provided a *Bauplan* is already given. Darwinism or the synthetic theory of evolution, however, are insufficient to explain macro-evolution, the origin of higher systematic units (Bertalanffy, 1929d:7; '37c:168; '52a:86). What is being challenged is the “nothing-but” claim that, in principle, this theory would be capable of providing a complete explanation of evolution (Bertalanffy, 1967:82; '69b:65). The reduced view of selectionists implies that every surviving structure or behavior has a selective advantage. On the one hand this is no proof that they were a product of selection (Bertalanffy, 1969ab:66). On the other hand this appears to be a questionable assumption. Thinking of selection solely in such terms seems to hinder asking further questions, since everything appears to be explainable. Selection would be the only directive agent, and thus it would be the environment and its changes that determine the course of evolution. Evolution would in that view be completely “outer-directed” (Bertalanffy, 1967:81; '69b:64)—a point of view that was also criticized by Uexküll (1973:286f).

Another critique is that selection already presupposes a self-maintaining system with certain characters and therefore can hardly explain them (Bertalanffy, 1928c:23; '67:82). Only because self-preserving organized systems are present, can selection act on them (Bertalanffy, 1932:59; '72:27). Random variations and selection appear insufficient for explaining the organic whole (Bertalanffy, 1929d:7; '32:59). The question arises if the hypothesis of mere random change hinders looking for laws of evolution (Bertalanffy, 1932:59). This is also an important and interesting issue for current research in evo-devo and evolutionary systems biology.

For Bertalanffy, the principle of selection is a limiting condition that increases the advantage of an organism; nonetheless, what is going on in special cases cannot be inferred from that principle. He illustrates this by an analogy to entropy. This also provides limiting conditions, and only provides a general direction. Entropy increases in closed systems. What happens in particular cannot be inferred from the principle of entropy; rather, specific conditions have to be considered (Bertalanffy, 1952a:93).

One further issue points to the insufficiency of the hypotheses on the causes of evolution in the synthetic theory. The ever increasing degree of organization cannot be explained by usefulness and adaptation (Bertalanffy, 1929d:7). Rather, it is an autonomous process caused by inner or internal factors that leads to this increase (Bertalanffy, 1929d:9; '51b:131). Internal factors are seen in contrast to external factors. The latter indicate an outer-directedness of evolution by selection (Bertalanffy, 1972:28; cf. also Schindewolf, 1936:92ff). The progression towards higher organization is a phenomenological fact. There is, however, neither proof that this has anything to do with adaptation, nor is there evidence that the conquest of new niches is connected to up-grading of the organizational level (Bertalanffy, 1967:83f; '69b:67). “The identification of evolution with adaptation is therefore by no means proved. It is a debatable point, not an *a priori* principle of evolution.” (Bertalanffy, 1969ab:68)

A key assumption in Darwinism—connected to the discussion on internal versus external factors—is the independence of one characters from the other, and correspondingly also of one gene from the other. This issue is connected to more recent accounts on burden (Riedl, 1977) or developmental constraints (cf. Wagner, 2014), which also question the idea of completely independent parts. From the perspective of Darwinism the organism is an aggregate of characters, and not a system: each part can be changed (by chance) without effecting the others (Bertalanffy, 1932:48; '52a:11). But for Bertalanffy the organism is more than a heap of hereditary characteristics or genes which are shuffled together by accident (Bertalanffy, 1955:254; '69a:237). The recurring example to clarify this point is the lens eye. In the course of evolution, changes in one part of an eye have to mesh with changes in other parts. A soft lens, a ciliary body, ciliary muscles and nerves must all be present and work together to enable its functioning (Bertalanffy, 1928a:163; '32:60; '52a:89f). This raises the question of co-adaptation, the harmonious change of the organization of an organism throughout evolution (Bertalanffy, '49b:81; '51a:345). The probability that independent changes in the single parts would lead to a functioning whole organ seems very low (Bertalanffy, 1952a:89f).

Certain empirical facts must be considered when examining the sufficiency of theories. In this regard Bertalanffy frequently points to parallelisms. Three kinds of parallelism indicate that chance is of limited importance (Bertalanffy, 1951a:208). First, changes in homologous genes can give rise to similar changes in different species; this is illustrated by Nikolai Vavilov's law of homologous series, according to which wheat and rye show parallel evolutionary stages. Second, similar phenotypes can arise from different genetic or environmental factors, for example the presence of albino forms in different species, some based on homologous, some on non-homologous genes. Third, parallelism can occur in spite of a different genetic and developmental basis; e.g., certain cases of mimicry (Bertalanffy, 1952a:98ff; '52b:165ff). Only particular trends seem to be allowed in evolution (Bertalanffy, 1952a:102). The formation of the lens eye—an example for the third type of parallelism—followed equal stages and paths in different classes of animals. Here, just one or a few “technical” solutions can satisfy the demands of a working organ. A parallelism is also evident in segmentation or in the evolution of circulatory systems in annelids and chordates (Bertalanffy, 1952a:102f; '52b:166f). Interesting in this regard is also that little change in physiology or basic chemicals is found in evolution, compared to huge morphological changes (Bertalanffy, 1928c:75; '32:134).

The conclusion from these considerations and findings is that “the changes undergone by organisms in the course of evolution do not appear to be completely fortuitous and accidental; rather they are restricted, first by the variations possible in the genes, secondly, by those possible in development, that is, in the action of the genic system, thirdly, by general laws of organization” (Bertalanffy, 1952a:103).

### Towards the Organismic Approach in the Theory of Evolution

Bertalanffy distinguishes two kinds of character: organizational characters (*Organisationsmerkmale*) which are a result of autonomous, inner or system processes; and adaptational characters (*Anpassungsmerkmale*) which result from a response to environmental conditions (Bertalanffy, 1933a:74). Accordingly, not all characters are advantageous or useful with respect to the environment (Bertalanffy, 1937c:171). Systematically important characters, such as leaf shape or number of vertebra, are among them (Bertalanffy, 1934:362; '52a:87).

Especially Karl Goebel ('28) pointed out that many plant organs can only be understood by means of **i**nner form laws (*innere Formgesetze*) and that such organs can then be utilized for different functions (Bertalanffy, 1933a:75; '37c:171). That only certain and not all configurations of inner connectedness are possible is reflected by analogous features (Bertalanffy, 1933a:75). Hence, Bertalanffy argues that more attention should be paid to analogies because they can help to find such inner, system or structural laws (Bertalanffy, 1937c:172; '69b:70). Studying analogies can shed light on evolutionary constraints which might be connected to the mentioned restrictions on the gene, developmental or organizational levels (Bertalanffy, 1967:85f). The analysis of Wake (1991) on whether analogies are a result of adaptation (natural selection) or limited developmental and structural options supports Bertalanffy.

In comparing crystals to organisms, Bertalanffy points out that both cannot be a product of mere random factors (Bertalanffy, 1952a:15). In the case of a crystal, forces are at work which do not allow any imaginable combination of the atoms. The crystal is in an equilibrium of forces that have already been investigated in physics: the results show that certain laws govern the phenomena. Similar organizing “forces” can possibly also be found in organisms and lead to only a few possible, even though highly complicated, equilibria (Bertalanffy, 1932:102,246; '72:27). This is related to more recent research on self-organization and evolution (cf. Hoelzer et al., 2006; Karsenti, 2008).

Bertalanffy assumes that species are not distinguished from each other by single genes, but rather by distinguished co-ordinations of genes (*Gengefüge*) (Bertalanffy, 1951a:347; '51b:132; '52b:164). Not the single genes are stable in this regard, but rather the organization of genes must be in a stable condition. Transitions from one such organization to another, viz from one species to another, are unstable and therefore not well preserved (Bertalanffy, 1946). “A ‘species' represents a state in which a harmoniously stabilized ‘genic balance' has been established, that is, a state in which the genes are internally so adapted to each other that an undisturbed and harmonious course of development is guaranteed.” (Bertalanffy, 1952a:96) “In a unitary conception of the basis of heredity, it may be possible to interpret phylogenetic changes not as an adding of new genes but rather as a transition to a new state in the genome as a whole” (Bertalanffy, 1952a:112). Related to this, Bertalanffy emphasized, in opposition to an aggregate view, that the whole genome generates (*hervorbringen*) the whole organism (Bertalanffy, 1934:359; '37c:161; cf. Woltereck, 1931:228). This contrasts with the Mendelian chromosome theory developed by T. H. Morgan in two ways. The American's theory is atomistic, ignoring interactions among genes, and no interest is shown in connecting genetics with development and evolution. The second critique was shared by many German geneticists, who considered Morgan's approach as valid but wanted to see a theory of inheritance that includes the role of genes in processes of development and evolution. In this way, their view was sharply distinguished from that of their American colleagues (Harwood, 1993:xv, 42f; cf. e.g., Woltereck, 1931:302; Schindewolf, 1936:87).

With regard to integrating genetics with development and evolution, Richard Goldschmidt has to be mentioned (Goldschmidt, 1927). His “principle of harmonized reaction-velocities” is acknowledged by Bertalanffy for providing an explanation for many processes in development such as: “progressive determination, organizer action, self-differentiation contrary to prospective significance (*bedeutungsfremde Selbstdifferenzierung*), polarity, development of bilateral asymmetry, compensatory growth, heteromorphosis, etc., and finally for the harmonization of growth rates both in its ontogenetic and phylogenetic aspects” (Bertalanffy, 1952a:62). The explanation via chemical reactions, however, does not solve the problem of “vital organization” of the developing system. Such an approach would shed light only on the production of chemically defined compounds, but not on the “organized formations” in an organism (Bertalanffy, 1932:324, '52a:62).

Genetics was seen by Bertalanffy as the field within biology that showed the highest state of progress, due to its exactness of laws and predictions. Gregor Mendel was not only an experimental biologist, but also an extraordinary theoretician (Bertalanffy, 1952a:69). For Bertalanffy, however, the organismic approach still needed to be introduced in genetics. For him all characters are polygenic, i.e., depending on the co-operation of many or all hereditary factors—yet with varying degrees of influence of single genes (Bertalanffy, 1952a:74). This approach is typical for Bertalanffy: He considers the possibility of connectedness of each part with every other, but does not deny that for certain characters only a few or even a single gene is decisive. A similar spectrum is seen by Bertalanffy for pleiotropy, where the role of one gene can vary from influencing a single character to influencing the whole organism. Due to this system property of genes influencing characters, the manifestation of one character can be influenced by many different factors. This goes beyond inherited factors to include factors from outside, such as temperature; which is unsurprising in view of the assumed catalytic nature of gene action. From a theoretical point of view the definition of a gene must be clarified when referring to the experimental basis. With respect to hybridization experiments, “a gene is not a unit or *Anlage* producing by itself a definite character or organ” (Bertalanffy, '52:75). Rather, a gene that is traceable in hybridizable organisms indicates the difference between two genomes that, as wholes, correspond to each other. Although Bertalanffy criticized Goldschmidt's approach, he acknowledged the far-reaching embryologic and phylogenetic consequences of the principle of harmonized reaction-velocities. Early developmental changes in the reaction rates, which are also connected to different rates of developmental processes, can lead to significant transformations, e.g., in shape. In that way the genome can be connected to developmental and evolutionary change (Bertalanffy, 1932:323, '52:78f).

Bertalanffy recognizes the possibility to find quantitative laws of evolution—the aim of theoretical biology—in allometric growth equations, as investigated by Julian Huxley ('32). Thus, the elongation of the horse's skull during one hundred million years of evolution seems to be governed by a simple arithmetic formula (Bertalanffy, 1951a:337; '52b:167f). Another indication of quantitative laws is the connection between body weight and basal metabolic rate, the so-called mouse-elephant curve (Bertalanffy, '60:142). The allometric constants of organs and biological functions are within the limits drawn by physical similarity when enlarged from small-scale to larger size (Bertalanffy, '60:228). This also provides insights into the issues of co-adaptation, harmonious transformation of the organism as a whole, and orthogenesis (Bertalanffy, '60:245). In such cases the organism is apparently governed by definite laws of the system as a whole (Bertalanffy, 1952b:167). Logically, one should—with regard to the organizational and adaptational characters—distinguish between allometric phenomena that are unfavorable and those that are advantageous.

Bertalanffy states that there are laws of organization which are often manifest as “evolutionary constraint” (Bertalanffy, '60:247). In this regard he acknowledges the work of Bernhard Rensch—a German zoologist who contributed to the modern synthesis and saw no need to introduce new principles beyond those that are at work in micro-evolution for explaining macro-evolution, Rensch “aptly speaks of *bionomogenesis*, i.e., evolution as the result of complex causal relationships of the environment as well as in the organism itself” (Bertalanffy, '60:247). He even honors Rensch on a birthday occasion (Bertalanffy, 1965). Indeed, Rensch considers laws of organization or systems (*Gefügegesetze*, *Systemgesetze*) and refers to Bertalanffy (cf. e.g., Rensch, 1968).

While Bertalanffy fully appreciates modern selection theory, he holds that evolution is co-determined by organic laws, which in suitable cases can be formulated precisely (Bertalanffy, 1952a:104f; '52b:164,168). Such laws of organization have to be formulated at each level, because at each level of organization new non-summative properties appear that cannot be obtained from isolated parts. In other words: there are principles of organization that do not require genetic control and, hence, cannot be the outcome of random mutations and selection (Bertalanffy, '69b:68f). Combining the different approaches might lead to a more profound insight into evolution. Interestingly, similar views to the organismic one seem to be taken by Ernst Mayr ('65), as Bertalanffy likes to point out (Bertalanffy, 1967:86f).

## HOMOLOGY

Unsurprisingly, Bertalanffy also applied his methods in theoretical biology to a central concept of evolutionary thought: homology. The historical and theoretical clarification of such terms is an important task within theoretical biology. Hence, Bertalanffy used this method in various fields and for various central concepts, even though they belonged to areas—such as evolution—that were not his primary focus. He wanted to show, however, that his organismic or system approach is useful for any biological area. He starts by analyzing the ideas brought forward so far and then introduces his own, organismic view on the concept of homology. He did this in depth in only one German article that appeared in a less well known journal (Bertalanffy, 1936; nonetheless, a translation was published posthumously: Bertalanffy, '75) and is interesting for evo-devo and evolutionary systems biology. Most of the following is taken from that '36 article.

Bertalanffy starts his analysis with the problems that the founding fathers of morphology—Goethe and Étienne Geoffroy Saint-Hilaire—addressed. In Goethe's morphology the single forms of organisms appear as different embodiments of an ideal type (*Typus*). This calls for clarifying the meaning of the often misunderstood term type. Type refers to an ideal image (*Urbild*) of different forms that never existed, not to be confused with ancestor organisms that really existed. The term, however, is not a mere product of fantasy. In modern terms, type would refer to a composition or organization law (*Aufbaugesetz*) of a group of entities similar to a general structural formula in chemistry. In this sense, a type or a general structural formula cannot be directly observed, but they are not “unreal” either. Hence, the term type can be used without any connotations of an idealistic Platonism. Logically, it has an equal status and is equally “real” as a law of nature: both are mental constructs and cannot be directly observed in nature. From a certain perspective the concept of type belongs to the descriptive stage, without any claim of explanation (cf. the stages of biology above). For an explanation, however, the governing laws have to be found (Bertalanffy, 1949b:80ff).

Throughout the course of history, Bertalanffy identifies three successive definitions of the homology concept. The first is what he terms the typological concept of homology (*typologischer Homologiebegriff*), which refers to Richard Owen: Homologous organs correspond to each other according to their location, irrespective of different functions. They are at the same position with respect to neighbor organs.

This is followed, second, by what slightly misleadingly is called the typologico-evolutionary concept of homology (*typologisch-entwicklungsgeschichtlicher Homologiebegriff*); typologico-developmental homology would be a more appropriate term: Homologous are those organs that develop from equal embryonic primordia (*Anlagen*); such a view was already present in Karl Ernst von Baer's thinking (Brigandt, 2011). This refers to the investigations of Karl B. Reichert on the development of the bones in the inner ear. Even though in adult individuals a homology based on neighboring parts is no longer valid, it still holds for the primordia. The typological concept is still basic.

The first two concepts were developed before the doctrine of descent (Darwin) was introduced. The theory of evolution consequently lead to the third concept, the phylogenetic concept of homology: Homologous are those organs that phylogenetically originate from each other or from a common ancestor. While some welcomed the new concept, not everyone was convinced and some challenged the autonomous value of this concept of homology. The phylogenetic concept did not replace the typological concept. Rather the typological concept—be it about the locations of organs in adult or embryonic primordia—constitutes an important criterion for the phylogenetic concept of homology. The view that homology has its value independent of evolutionary interpretation was broadly acknowledged by evolutionary morphologists (Russell, 1916:355, Brigandt, 2011). Location is directly observable, descent is not. Morphology thus appears as one basis for finding evolutionary explanations. The debate whether morphology should be conducted without considering phylogeny or not continued into the 20th century; cf. e.g., the different views of Adolf Naef, Willi Hennig, and others (Rieppel, 2006, 2012; see also Schindewolf, 1928 for the relationship of systematics and phylogeny). Note, however, that Bertalanffy perceives morphology as one means amongst others to clarify the course of evolution. Morphology includes no time dimension, whereas other aspects, such as stratigraphy, do. Hence, “not only does the typological homologization serve as the base for the phylogenetic derivation, but vice versa, the morphological clarification of uncertain elements is often possible only by means of a phylogenetic analysis” (Bertalanffy, '75:91). This is a typical instantiation of Bertalanffy's perspectivist epistemology (Pouvreau and Drack, 2007:289ff).

The concept of homology was also challenged by developmental biologists. The regeneration of the eye lens, after operational removal, shows that it does not develop from the epidermis, as in normal development, but from the side of the iris. What does that mean for the (second) concept of homology? From equal material, different organ parts can develop, and different material can develop into equal parts, such as a lens. For Hans Spemann (1915)—who is well known for his transplantation experiments and the conception of embryonic induction by organizers—the homology concept seemed to dissolve (cf. Laubichler, 2000). Also Gavin Rylands de Beer, who was the first to note that the sameness of genes and sameness of morphological structures do not map, criticized the traditional embryological criterion of homology (Brigandt, 2006). For Bertalanffy, however, the difficulties arise from preconceived opinions, and he asks for a new conception because, in normal development, such artificial experiments do not occur.

The old homology concepts were preformistic to Bertalanffy. Underlying these concepts is the idea that certain primordia for certain formations are already there in the beginning. Developmental biology, however, shows a non-preformistic and epigenetic character. Epigenesis refers to the interaction of the parts in the whole system (Bertalanffy, 1937c:107). What is fixed at the beginning is not the material primordia of the single organs, but rather the organizational relationships that brings forth a species-specific organism. Within the organizational relationships the formation of the lens is bound to a specific location and is independent of the material. The homology concept can thus be maintained if the essential factor is not the material from which a particular organ is formed, but rather the organizing relations. The old concept of homology of equal locations still holds if, by “location,” a dynamically effective factor rather than a reference to geometric site is meant.

Bertalanffy therefore defines a developmental physiology (*entwicklungsphysiologisch*) or dynamic concept of homology: Homologous are those organs that developed in equal location, i.e., under according organizational relationships; or: “organs which occupy the same position in the organizational set of relations” (Bertalanffy '75:95). Equal location remains basic, yet no longer in the sense of an ideal relationship but rather in a “real” organizational causal relationship. Not the static form is essential, but rather the site of certain system conditions. This concept of homology reflects the fact that organic form can only be conceived of as being dynamic. Even though Bertalanffy provides no more than a sketch of a new homology concept, it is related to modern, more detailed accounts that investigate the causal basis of homology (Wagner, 1989; 2014).

Note that one critique of Bertalanffy's analysis of the homology concept came from Adolf Remane. To clarify the theoretical foundations of phylogeny and systematics, the latter sought an understanding of the principles of comparative anatomy and, hence, an operational account of the homology concept (Laubichler, 2000; Brigandt, 2006). Just before Remane describes his well-known criteria of homology, he notes that since there is only one natural system (*natürliches System*) there can only be one concept of homology. The different concepts of homology, as described by Bertalanffy, would rather refer to partial criteria of a single concept of homology (Remane, 1952:32f). That critique is not well justified. Remane himself points out that the concept was gradually developed. Importantly, Bertalanffy provides precisely this overview and analysis of the meaning different authors gave to the term throughout history, and holds that location is the most important criterion (Bertalanffy, 1937c:166). Importantly, Bertalanffy was interested in the causal basis of homology and not so much in operational criteria for distinguishing between homologies and non-homologies.

Bertalanffy's new conception of homology is a typical example of how he used the dynamic systems or organismic approach to reinterpret established facts and therewith made progress in uniting knowledge form different threads of research.

## CONCLUSION

Bertalanffy's approach can be seen as opening the field of potential explanations. He articulates the basic problems of biology and relates them to potential explanatory pathways. The general phenomena of life are connected to the organization of the parts and processes within an organism. Considering the regularities in such system behaviors prompts Bertalanffy to propose research designed to discover the laws that govern them. The organismic approach is—without neglecting the merits of purely analytical endeavors—important for all fields of biology, and hence also for evolutionary biology and even the concept of homology.

Bertalanffy had an effect on many researchers in different fields, but probably only a few evolutionary biologists were directly influenced by him. One of his students was Rupert Riedl, who is also prominently quoted by Gould and Lewontin (1979). The influence of organismic biology or the system theoretic approach is clearly evident in Riedl's work (Riedl, 1977, 2000). Based on system thinking, Riedl derived a theory that reduces the role of chance in evolution and hence the role of mere adaptation, leading to the notion of burdens that constrain the course of evolution.

To summarize Bertalanffy's approach, one could alter a famous statement by Dobzhansky (1973) and say: Nothing in biology makes sense except in the light of organization. This is true even for evolution.
